# The Long-Term Effect of Intensity Modulated Radiation Therapy for Prostate Cancer on Testosterone Levels

**DOI:** 10.1016/j.adro.2021.100851

**Published:** 2021-11-17

**Authors:** Yutaka Horiguchi, Fumio Tsukuda, Ayato Ogata, Kiichi Hagiwara, Noboru Sakamoto, Yukihiro Hama, Shoji Koga

**Affiliations:** a Department of Urology, Edogawa Hospital, Tokyo, Japan; b Department of Radiology, Edogawa Hospital, Tokyo, Japan

## Abstract

**Purpose:**

Concern about a long-term effect of the delivery of intensity modulated radiation therapy (IMRT) for prostate cancer on serum testosterone levels remains unelucidated. We evaluated how IMRT for localized prostate cancer affects serum testosterone levels during a follow-up period of up to 10 years.

**Methods and Materials:**

We retrospectively evaluated data from 182 patients with localized prostate cancer who underwent definitive IMRT alone between 2007 and 2014. Serum total testosterone (TT) levels were measured by blood draws between 6 AM and 11 AM before treatment and at every posttreatment follow-up for 10 years. Pretreatment values and each posttreatment testosterone value were compared using a Wilcoxon signed rank test. The data set was stratified into 4 groups based on the pretreatment testosterone (pre-TT) values using quartiles.

**Results:**

The median absolute or relative changes in TT levels from pretreatment were –0.42 ng/mL or –12.0% at 3 months after radiation therapy (*P* < .0001). Subsequently, TT levels gradually recovered to nearly the pretreatment levels 24 to 36 months after IMRT. When analyzed according to the pre-TT quartile, median TT levels initially decreased at the 3- to 12-month period in all the quartiles; however, median TT levels increased from the 18-month period in the first and second quartile groups, whereas they were maintained at less than the pretreatment levels in the third and the fourth quartile groups throughout the entire decade after radiation therapy. The proportion of patients with hypogonadal status, defined as TT levels <3.00 ng/mL, did not increase over time.

**Conclusions:**

A transient and modest decrease of TT levels after IMRT spontaneously recovered to the pretreatment levels at the 24- to 36-month period except in patients in the higher quartile of pre-TT. This might have been partly owing to a variable sensitivity of individual testicular function to scattered radiation. Patients with lower pre-TT did not demonstrate a progressive overall rate of hypogonadism until 10 years after radiation therapy.

## Introduction

Previous studies on external-beam radiation therapy (EBRT) have evaluated changes in serum testosterone levels in patients receiving EBRT for prostate cancer^1^, ^2^, ^3^, ^4^, ^5^, ^6^, ^7^, ^8^, ^9^ and other pelvic malignancies.^10^^,^^11^ NRG Oncology, a newly developed clinical trials network group consisting of the coordinated efforts of the National Surgical Adjuvant Breast and Bowel Project, the Radiation Therapy Oncology Group (RTOG), and the Gynecologic Oncology Group recently reported on the RTOG 9408 randomized clinical trial and found that a total dose of 68.4 Gy to the prostate was only associated with a median decrease in testosterone of 13.5% at 3 months after treatment.^7^ The majority of previous studies have also shown that patients experienced only a transient decrease of serum testosterone levels after EBRT.^2^^,^^4^, ^5^, ^6^^,^^8^ Furthermore, low-dose scattered radiation to testicular Leydig cells is believed to be the most likely explanation for this phenomenon. However, the data either were from a database of past clinical trials, and thus testosterone follow-up was not unified,^7^^,^^8^ or were acquired from a limited number of patients.^3^^,^^5^^,^^6^ Moreover, one study has reported that scattered testicular radiation does not play a significant role in the reduction of the serum testosterone level.^2^ These limitations and inconsistencies raise a concern regarding a long-term effect of radiation on testosterone levels. Thus, this study aimed to evaluate the long-term effect of intensity modulated radiation therapy (IMRT) on serum testosterone changes in patients with localized prostate cancer. Toward this end, we examined changes in serum total testosterone (TT) levels and whether the associated risk of biochemical hypogonadism increased owing to the incidental scattered dose to the testes exposed during the treatment.

## Methods and Materials

### Cohort of patients and follow-up

This was a retrospective study of patients with localized prostate adenocarcinoma without any radiologic evidence of distant metastasis (any T, N0, M0) who received definitive IMRT monotherapy without any neoadjuvant or adjuvant hormone therapy between June 2007 and December 2014. Elective pelvic nodal radiation was not delivered. Of the 615 eligible patients, we excluded 33 patients with missing pretreatment TT (pre-TT) values; to avoid the known diurnal variability of TT levels, we also excluded 400 patients whose pre-TT values were determined by blood draws that took place after 12 PM.^12^ Thus, data from 182 patients were included in this study. These 182 patients were stratified according to the pre-TT levels per quartile (pre-TT Q1-Q4), and changes in TT levels of each group were evaluated over time.

Altogether, 181 patients (99.5%) received a total dose of 76 Gy IMRT at 2 Gy per fraction with 6 million electron-volt (MV) photons to the prostate gland. The original dose of 76 Gy was reduced to 72 Gy in a single patient owing to the patient's concerns regarding radiation-related complications.

Prostate-specific antigen (PSA) recurrence was defined based on the Phoenix criteria^13^ or the initiation of second-line treatments. The TT levels were measured during the following scheduled periods: immediately before the IMRT, 3 and 6 months after IMRT completion, at every 6-month follow-up visit thereafter for 3 years, and once a year thereafter until 10 years after IMRT. The TT concentration was measured using a commercial solid-phase radioimmunoassay (SRL, Inc, Tokyo, Japan). The TT levels based on blood draws after 12 PM at each scheduled period were excluded from the study. Thus, the percentage of patients excluded from the study at each time point ranged from 20.6% (34 of 165 patients at 3 months) to 47.7% (62 of 130 patients at 72 months). A hypogonadal TT level was defined as <3.00 ng/mL in accordance with the testosterone deficiency guideline of the American Urologic Association.^14^

### Statistical analysis

A Wilcoxon signed rank test or Kruskal-Wallis test was used to compare changes in pretreatment and posttreatment TT values. All statistical tests were performed using SPSS Statistics, version 25 (IBM Corporation, New York, New York), and *P* < .05 was considered statistically significant.

This study was approved by the institutional ethics review board of Edogawa Hospital and carried out in accordance with the Declaration of Helsinki, and all the patients provided informed consent before participating in the study.

## Results

The median follow-up period was 96 months (range, 7-148 months). Recurrence of PSA occurred in 31 patients (17.0%) within a median of 63 (14-121) months after IMRT. The overall 5- and 10-year PSA recurrence-free survival was 91.0% and 78.6%, respectively. None of the patients subsequently received any kind of hormone therapies until PSA recurrence was evident, at which point the TT levels of those patients with hormone therapies were excluded from the database of the study. The characteristics of the 182 patients stratified according to their pre-TT levels per quartile (pre-TT Q1-Q4) are shown in Table 1. There were no significant differences in terms of age, body mass index, diabetes mellitus, smoking habits, alcohol consumption, and comorbidities or in the time of blood draw between the 4 quartile groups. There were also no significant differences with respect to oncological background including initial PSA level, tumor stage, biopsy-proven pathologic Gleason score, and rate of PSA recurrence.

**Table 1:** Characteristics of eligible patients stratified by the pre-TT levels per quartile (N = 182)

		Serum testosterone levels at pretreatment, ng/mL				
	Overall	Q1 (<3.37)	Q2 (≥3.37 to <4.23)	Q3 (≥4.23 to <5.43)	Q4 (≥5.43)	
Patients, no.	182	46	45	45	46	*P* value*
						
Time of blood draw, AM						.959
Median	10:39	10:35	10:47	10:36	10:34	
IQR	10:03-11:08	10:08-11:05	10:11-11:08	10:01-11:23	10:02-11:11	
Age, y						.786
Median	71	70	71	72	71	
IQR	67-75	66-74	67-75	67-75	68-76	
BMI, kg/m^2^						.103
Median	23.6	24.5	23.2	23.5	23.3	
IQR	22.0-25.6	23.1-26.5	22.0-25.5	21.6-25.7	21.8-24.7	
Diabetes mellitus						.378
Yes	26 (14.3%)	8 (17.4%)	9 (20.0%)	5 (11.1%)	4 (8.7%)	
No	156 (85.7%)	38 (82.6%)	36 (80.0%)	40 (88.9%)	42 (91.3%)	
Smoking status						.774
Yes	50 (27.5%)	14 (30.4%)	14 (31.1%)	10 (22.2%)	12 (26.1%)	
No	111 (61.0%)	29 (63.0%)	24 (53.3%)	27 (60.0%)	31 (67.4%)	
N/A	21 (11.5%)	3 (6.5%)	7 (15.6%)	8 (17.8%)	3 (6.5%)	
Alcohol consumption						.815
Yes	103 (56.6%)	30 (65.2%)	26 (57.8%)	21 (46.7%)	26 (56.5%)	
No	58 (31.9%)	13 (28.3%)	15 (33.3%)	14 (31.1%)	16 (34.8%)	
N/A	21 (11.5%)	3 (6.5%)	4 (8.9%)	10 (22.2%)	4 (8.7%)	
Comorbidities						.347
Yes	118 (64.8%)	30 (65.2%)	32 (71.1%)	31 (68.9%)	25 (55.6%)	
No	64 (35.2%)	16 (34.8%)	13 (28.9%)	14 (31.1%)	20 (44.4%)	
Tumor stage						.182
<T3	147 (80.8%)	38 (82.6%)	36 (80.0%)	32 (71.1%)	41 (89.1%)	
≥T3	35 (19.2%)	8 (17.4%)	9 (20.0%)	13 (28.9%)	5 (10.9%)	
Gleason score						.207
<4 + 3	116 (63.7%)	30 (65.2%)	30 (66.7%)	23 (51.1%)	33 (71.7%)	
≥4 + 3	66 (36.3%)	16 (34.8%)	15 (33.3%)	22 (48.9%)	13 (28.3%)	
PSA level, ng/mL						.417
Median	7.54	6.94	7.88	7.14	8.46	
<10	125 (68.7%)	31 (67.4%)	31 (68.9%)	34 (75.6%)	29 (63.0%)	
≥10	57 (31.3%)	15 (32.6%)	14 (31.1%)	11 (24.4%)	17 (37.0%)	
Prostate volume, cm^3^						.300
Median	30.8	29.6	32.0	34.2	27.5	
IQR	24.1-39.0	25.6-38.4	25.0-41.0	26.0-41.9	22.8-36.6	
Radiation dose, Gy						.385
72	1 (0.5%)	0 (0%)	0 (0%)	1 (2.2%)	0 (0%)	
76	181 (99.5%)	46 (100%)	45 (100%)	44 (97.8%)	46 (100%)	
Testosterone level, ng/mL						<.0001
Median	4.23	2.81	3.91	4.73	6.02	
IQR	3.37-5.43	2.52-3.13	3.71-4.07	4.44-5.09	5.77-6.67	
PSA recurrence						.377
Yes	31 (17.0%)	5 (10.9%)	8 (17.8%)	11 (24.4%)	7 (15.2%)	
No	151 (83.0%)	41 (89.1%)	37 (82.2%)	34 (75.6%)	39 (84.8%)	
Observation period, mo						.367
Median	95.6	93.0	100.1	93.4	94.6	
IQR	80.1-114.3	67.7-114.2	91.1-119.4	81.1-113.3	80.9-112.1	

*Abbreviations:* BMI = body mass index; IQR = interquartile range; N/A = not available; PSA = prostate-specific antigen; Q1-Q4 = first, second, third, and fourth quartile; TT = total testosterone.

⁎ Kruskal-Wallis test.

### Changes in testosterone levels

**Fig. 1:**
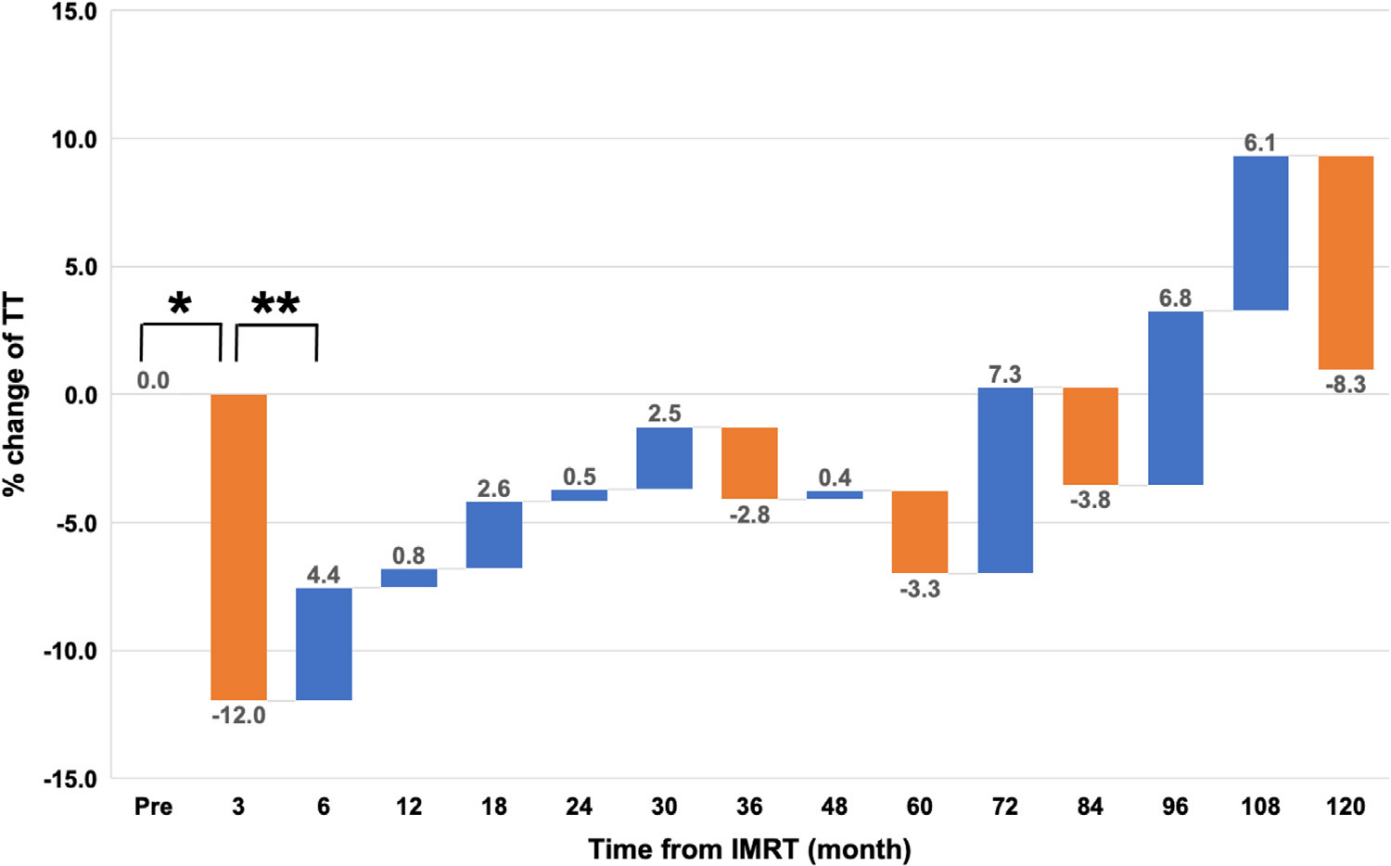
Percent changes at each interperiod (median). The transient decrease (–12.0%) in TT levels was observed only once, immediately after IMRT at the 3-month period (Wilcoxon signed rank test: *P < .0001; ** *P* < .001). *Abbreviations:* IMRT = intensity modulated radiation therapy; TT = total testosterone.

**Fig. 2:**
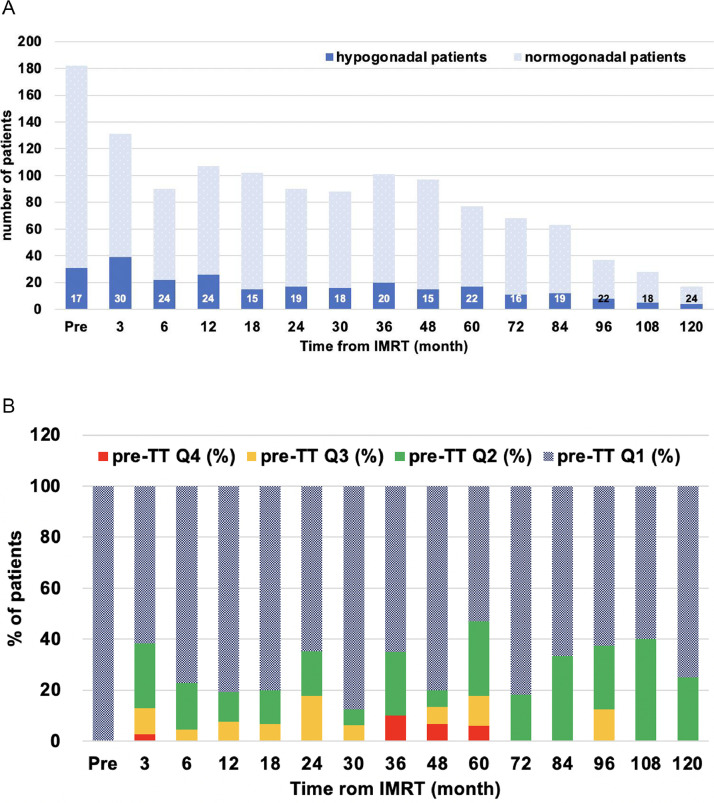
A, Proportion of patients with hypogonadal status (TT level, <3.00 ng/mL) over time until 10 years after IMRT. The median age of patients and percentage of hypogonadal patients, respectively, at each time point are shown at the top and bottom of the bar chart. B, Percentage distribution of pre-TT quartile 1 to quartile 4 patients among hypogonadal patients. *Abbreviations:* IMRT = intensity modulated radiation therapy; TT = total testosterone.

The TT levels of all 182 patients at each period are summarized in Table 2. Data on TT levels at 3 months after IMRT were available in 131 patients. The median absolute and relative changes in TT from pre-TT levels of these 131 patients were significantly lower (median, –0.42 ng/mL and –12.0%; both *P* < .0001). The subsequent absolute and relative changes in TT values at each period are shown in Table 2. There was a significant decline in TT levels from 3 to 12 months after IMRT, with the peak relative changes observed at 3 months (–12.0%). The TT levels then gradually increased and reverted closer to the pre-TT levels at 24 to 36 months after IMRT. We also evaluated the changes in TT at each “intertime” period in the overall cohort and found that the transient decrease in TT levels was observed only once immediately after IMRT (median, –12.0% at the 3-month period; *P* < .0001; Fig 1). Subsequently, from the 6-month period, TT levels significantly increased (*P* < .001) until the 30-month period (Fig 1). In total, the proportion of patients with hypogonadal TT levels was nearly equal between the 30-month period (16 of 88 patients [18.2%]) and the pretreatment period (31 of 182 patients [17.0%]). As shown in Figure 2A, a proportion of patients with hypogonadal status remained at the same level over time until 10 years after IMRT. Patients with pre-TT Q2 to Q4 constituted 13% to 47% of the overall hypogonadal patients at each period (Fig 2B).

**Table 2:** Total testosterone changes

Post-IMRT, mo	Pre	3	6	12	18	24	30	36	48	60	72	84	96	108	120
Patients, no.	182	131	90	107	102	90	88	101	97	77	68	63	37	28	17
															
TT levels, ng/mL															
Median	4.23	3.85	3.98	3.86	4.31	4.34	4.29	3.92	4.27	4.11	4.26	4.28	4.21	4.57	3.77
IQR	3.37-5.43	2.86-4.62	3.02-4.88	3.07-5.05	3.48-5.30	3.44-5.22	3.44-5.41	3.09-5.49	3.41-5.28	3.26-5.12	3.42-5.47	3.42-5.48	3.41-5.09	3.37-5.43	3.01-4.65
*P value	-	<.0001	<.001	<.01	NS	<.05	NS	NS	NS	NS	NS	NS	NS	NS	NS
											
Absolute changes of TT from pretreatment level, ng/mL															
Median	0.00	-0.42	-0.26	-0.25	-0.19	-0.23	-0.13	-0.05	-0.13	-0.30	-0.33	-0.12	-0.30	0.08	-0.33
IQR	0.00-0.00	-1.20 - 0.10	-0.90 - 0.10	-1.01 - 0.24	-0.90 - 0.66	-1.01 - 0.43	-0.82 - 0.58	-0.78 - 0.44	-0.66 - 0.59	-1.06 - 0.67	-0.81 - 0.39	-0.85 - 0.89	-0.94 - 0.68	-0.78 - 1.06	-1.07 - 0.71
*P value	-	<.0001	<.001	<.01	NS	<.05	NS	NS	NS	NS	NS	NS	NS	NS	NS
											
Relative changes of TT from pretreatment level, %															
Median	0.0	-12.0	-6.5	-7.4	-4.6	-6.0	-4.0	-1.3	-2.7	-7.5	-6.2	-3.7	-8.1	2.1	-13.0
IQR	0.0-0.0	-26.8 - 2.3	-19.7 - 2.2	-21.4 - 5.2	-20.6 - 17.8	-20.1 - 10.8	-19.3 - 18.1	-16.2 - 10.0	-14.8 -18.2	-24.1 - 15.2	-19.5 - 8.8	-20.7 - 19.1	-19.6 - 16.1	-15.5 - 23.6	-20.5 - 16.6
*P value	-	<.0001	<.01	<.01	NS	NS	NS	NS	NS	NS	NS	NS	NS	NS	NS

*Abbreviations:* IMRT = intensity modulated radiation therapy; IQR = interquartile range; NS = not significant; TT = total testosterone.

⁎ The Wilcoxon signed rank test compared the pre-TT value with corresponding values at each period.

### Stratified analysis based on pretreatment testosterone levels

**Fig. 3:**
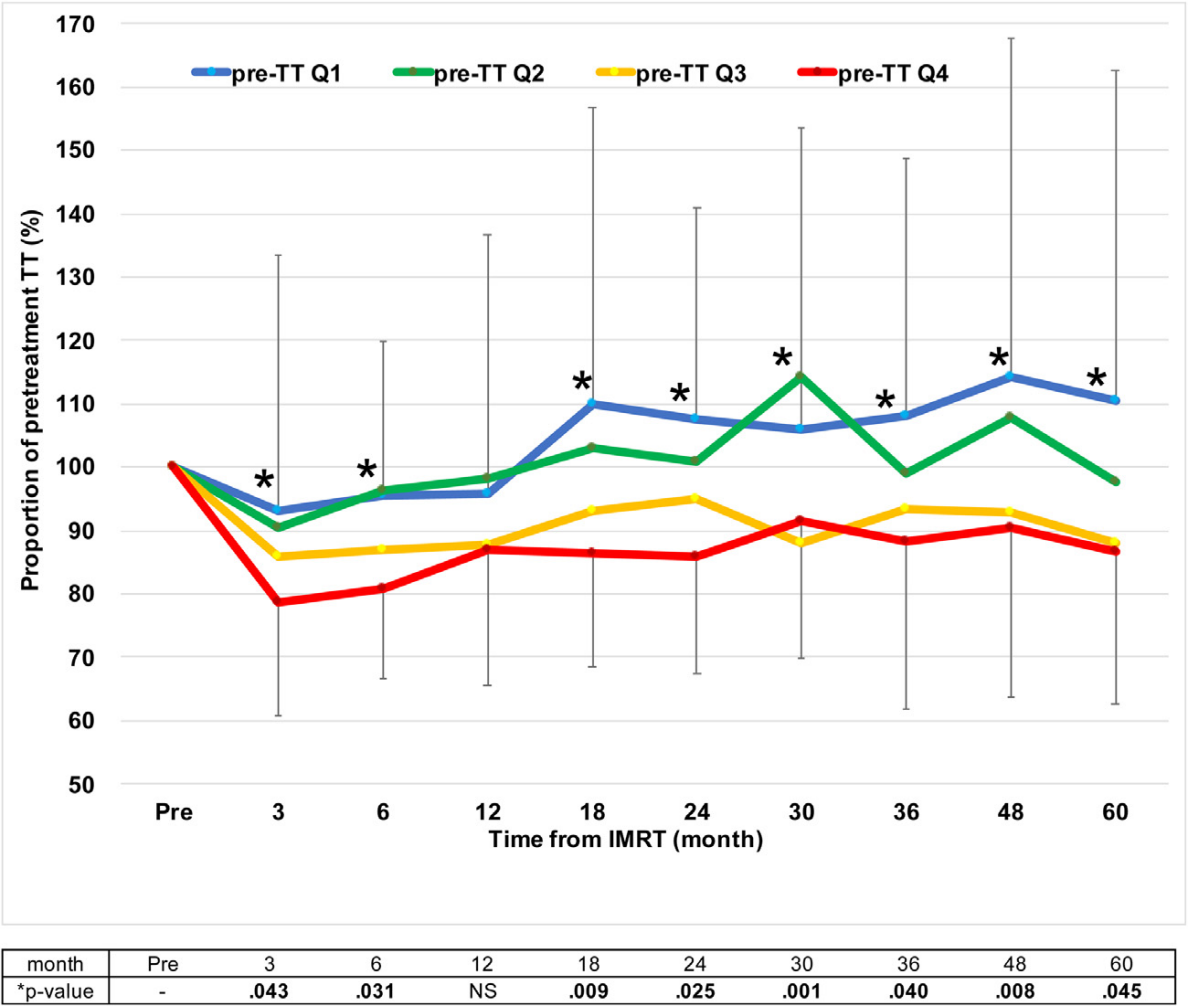
Proportional change from pretreatment TT level at each period. The proportional changes from pre-TT levels at each period were significantly different among the 4 quartile groups until the 60th month (*statistically significant; Kruskal-Wallis test). *Abbreviations:* IMRT = intensity modulated radiation therapy; NS = not significant; TT = total testosterone.

When analyzed according to the pre-TT quartile (pre-TT Q1-Q4), median TT levels initially decreased at the 3- to 12-month period in all the quartiles; however, median TT levels increased from the 18-month period in the first and the second quartile groups (pre-TT Q1 and Q2; Table 3). In contrast, median TT levels were significantly suppressed through most of the observed period until the 72nd month in the third and the fourth quartile groups (pre-TT Q3 and Q4; Table 3). Throughout the observed periods until the 60th month, other than the 12th month, the proportional changes from pre-TT levels at each period were significantly different among the 4 quartile groups (Fig 3).

**Table 3:** Relative percentages of TT level

Post-IMRT, mo	Pre	3	6	12	18	24	30	36	48	60	72	84	96	108	120
Pre-TT Q1															
Patients, no.	46	36	23	29	24	21	24	26	26	17	14	13	7	7	6
Median	100.0	93.0	95.5	95.7	109.9	107.5	106.0	108.1	114.2	110.6	98.0	78.9	89.5	117.8	97.3
IQR	100.0-100.0	80.4-116.1	86.7-114.4	87.8-128.4	86.7-143.9	89.2-115.0	89.3-122.0	92.6-122.0	92.8-139.7	100.7-131.8	80.8-113.0	62.3-130.0	77.1-114.3	88.7-134.0	86.1-118.0
*P value (pre vs each time)	-	NS	NS	NS	NS	NS	NS	NS	NS	NS	NS	NS	NS	NS	NS
Pre-TT Q2															
Patients, no.	45	31	24	21	21	20	20	24	25	21	20	23	12	10	5
Median	100.0	90.5	96.4	98.1	102.9	100.9	114.3	98.9	107.7	97.5	99.1	109.6	99.5	102.1	83.0
IQR	100.0-100.0	75.1-109.0	84.0-103.7	84.8-117.1	89.3-120.8	85.7-119.4	102.8-127.2	82.6-109.2	94.7-125.6	75.3-121.3	84.2-117.7	89.7-127.6	88.1-121.3	83.7-138.6	73.8-102.4
*P value (pre vs each time)	-	NS	NS	NS	NS	NS	.011	NS	NS	NS	NS	NS	NS	NS	NS
Pre-TT Q3															
Patients, no.	45	30	20	30	30	26	19	25	22	19	15	14	10	7	4
Median	100.0	85.9	87.0	87.9	93.0	95.1	88.1	93.4	92.8	88.2	91.3	91.9	89.9	93.7	85.1
IQR	100.0-100.0	75.0-94.8	81.2-101.1	73.9-100.3	79.3-106.5	80.9-104.9	78.2-95.8	86.5-107.1	80.5-101.9	75.8-97.9	82.2-105.8	83.9-105.3	81.8-102.3	86.5-117.1	78.1-106.2
*P value (pre vs each time)	-	<.0001	.040	<.001	NS	NS	.023	NS	.033	.018	NS	NS	NS	NS	NS
Pre-TT Q4															
Patients, no.	46	34	23	27	27	23	25	26	24	20	19	13	8	4	2
Median	100.0	78.7	80.9	87.0	86.4	85.8	91.6	88.3	90.5	86.6	86.6	95.6	98.8	87.8	98.2
IQR	100.0-100.0	69.0-97.3	77.7-98.9	70.2-103.8	74.4-98.4	76.0-92.9	74.6-101.0	69.4-106.7	67.4-103.0	70.5-103.2	73.2-100.6	85.9-110.3	79.8-127.0	86.2-104.7	89.0-107.4
*P value (pre vs each time)	-	<.0001	<.001	.026	.003	.004	.030	NS	.027	.030	.040	NS	NS	NS	NS

*Abbreviations:* IMRT = intensity modulated radiation therapy; IQR = interquartile range; NS = not significant; Q1-Q4 = first, second, third, and fourth quartile; TT = total testosterone.

⁎ Kruskal-Wallis test.

## Discussion

This study evaluated the effect of IMRT for localized prostate cancer on serum testosterone changes during long-term follow-up. We found that IMRT for prostate cancer was associated with a median 12.0% reduction in testosterone levels 3 months after treatment. Further, testosterone suppression was only transient and significantly depended on the pretreatment levels of testosterone. To our knowledge, this is the first and largest study to evaluate long-term serial TT changes in patients receiving definitive IMRT for localized prostate cancer. Our results are consistent with those of previous studies that demonstrated transient TT depression immediately after photon irradiation for prostate cancer.^2^, ^3^, ^4^, ^5^, ^6^, ^7^, ^8^ However, to our knowledge, no previous study has reported statistically significant changes in posttreatment TT values among patients stratified by pre-TT levels. Notably, in this study, the median TT levels of patients with pre-TT values in the lowest quartile (pre-TT Q1, TT <3.37 ng/mL) never decreased significantly after the 3-month IMRT follow-up period. Markovina et al reported that patients with a normal baseline testosterone level (≥2.41 ng/mL) had, on average, a 21.2% larger relative decrease in testosterone than that seen in patients with a low baseline testosterone level (<2.41 ng/mL) at the 6-month time point (*P* = .03).^6^ However, the study included a limited number of patients (51, of whom 37 underwent definitive EBRT only), and the observation period was short (24 months after IMRT, at most). A study by Yuan et al, which included 636 patients with prostate cancer treated with stereotactic body radiation therapy who were observed for up to 24 months, showed that the testosterone level tended to increase in patients in the first tertile group of baseline testosterone.^9^ Yuan et al reported that patients with higher baseline levels of testosterone experienced a decline over time. However, they concluded that the decline was of unclear clinical significance because no patient reached hypogonadal or castrate levels of testosterone. Moreover, these previous studies, which found increased posttreatment testosterone levels in patients with a lower pretreatment level, did not discuss this phenomenon.^6^^,^^9^ A similar observation was reported by Taira et al, who evaluated testosterone changes after Pd-103 brachytherapy with low-dose EBRT (20-45 Gy) in selected patients with intermediate- or high-risk prostate cancer.^15^ They showed that men with a higher pretreatment testosterone level (>3.61 ng/mL) tended to experience a decrease in testosterone (*P* < .001), whereas men with an average or below average baseline testosterone level had no significant change. The authors discussed that the lack of testosterone suppression in their cohort could be explained partly by the very low radiation scatter dose to the testicles from brachytherapy, which they estimated to be 0.02 Gy over the life of an implant.

Some studies have previously attempted to estimate the amount of scattered radiation to the testes in the setting of prostate IMRT.^16^^,^^17^ Clinical investigations by King et al using thermo-luminescent dosimeter measurements estimated testicular scatter doses of 0.68 Gy from internal photon scatter for prostate-only fields of 6 MV.^16^ From daily fiducial image guidance, the testes-in-field mean dose was 3.50 Gy, whereas the testes-out-of-field scatter dose was 0.16 Gy for a complete IMRT course of 39 fractions. The authors concluded that incidental doses to the testes from prostate IMRT can be minimized by (1) opting to restrict the use of elective pelvic nodal fields, which would increase the testicular dose to 1.72 Gy for 6 MV energies; (2) choosing photon energies less than 10 MV; and (3) using the smallest port sizes necessary for daily image guidance.^16^ As such, each time IMRT needs to be performed, a computed tomography (CT) image is taken first to accurately locate the prostate for radiation. The CT scan taken at the time of initial treatment planning is then superimposed on the CT scan taken immediately before each treatment, and the position of the patient is accurately adjusted to achieve precise treatment. This procedure results in additional radiation exposure to the testes. The radiation dose exposure would be more than 21 times higher when the testicles are in the field of CT scanning. Thus, in the setting of definitive IMRT (76 Gy via 38 fractions with 6 MV energies to the prostate gland), depending on the specific treatment scenarios, it is feasible to deliver cumulative incidental mean testicular doses ranging from 0.84 Gy to 4.18 Gy.^16^

Compared with the germ cells, Leydig cells are relatively resistant to the effects of radiation.^18^ Recently, Faria et al published the results of their phase 1 study to achieve testicular castration induced by direct radiation to the whole scrotum.^19^ They tested 17 Gy in 2 fractions for 3 patients with advanced prostate cancer. However, all of the patients maintained normal levels of testosterone throughout the entire follow-up period until 36 months after radiation therapy. Compared with the current study, having estimated the exposed radiation dose in the treatment setting to range from 0.84 Gy to 4.18 Gy, the findings by Faria et al are striking; in their study, the authors used a 4.1 to 20.2 times higher treatment dose without causing testicular castration. This comparison might be carefully accounted for by the fact that the method associated with testes exposure to radiation in the study by Faria et al was completely different from that in our current study on conventional IMRT (ie, a total of 17 Gy in 2 fractions vs a maximum of 4.18 Gy in 38 fractions in our case).

The transient decline in testosterone is consistent with the findings of most studies on photon-based radiation therapy for prostate cancer. The current consensus is that low-dose scatter radiation outside of the beam path has a deleterious effect on testicular Leydig cell function.^3^, ^4^, ^5^, ^6^, ^7^, ^8^, ^9^ Rowley et al have shown that Leydig cell function was dose-dependently disturbed and later recovered through direct radiation exposures (up to 6 Gy) to the testis.^20^ Consequently, it is reasonable to consider that the higher activity of baseline Leydig cell function (and a higher pre-TT level) can increase sensitivity to radiation.^21^, ^22^, ^23^ This understanding might account for the contrasted changing patterns of posttreatment TT levels between pre-TT Q1 and Q4.^6^^,^^9^ Thus, we believe the mechanism underlying the different pattern in TT change may be owed at least partly to a potential gradation of individual sensitivity when Leydig cells are exposed to low-dose scattered radiation during approximately 7 weeks (5 times a week) of IMRT.

Kubo and Shipley reported that a gonadal shield and scrotal block significantly reduced the photon scatter dose to the testes to less than 0.1% of the prescribed midplane dose during retroperitoneal therapy with 10 MV x-ray.^24^ Although the use of shielding block was originally integrated into clinical practice for younger men with testicular cancer, Kubo and Shipley's findings suggest that it may also be beneficial for patients with prostate cancer.

In the current study, the standard IMRT for prostate cancer did not induce significant hypogonadal status overall; however, a cautious follow-up of sexual function as well as clinical management and counseling are recommended, especially in patients with TT levels equal to or more than 4.23 ng/mL.

This study has some limitations. First, TT levels are known to be susceptible to diurnal variation. Our findings were based on TT levels determined between 6 AM and 11 AM. Despite the inherent bias in this retrospective study, the proper sample size, strictly excluding the data derived from blood draws after 12 PM, ensures robustness of the present findings. Second, we did not evaluate the adverse effect of IMRT on testicular function. We made every effort to correlate changes in TT levels with changes in sexual function according to the International Index of Erectile Function questionnaire sheet. Because this was a retrospective observational study, the limited number of patients with data on sexual function (46 of 182 [25.3%], data not shown) precluded a more in-depth statistical evaluation. Lastly, the current study lacked a control arm without radiation effects. It would be ideal to include an age-matched group of patients with prostate cancer treated with other modalities such as surgery to compare spontaneous changes in testosterone levels together with luteinizing hormone and follicle stimulating hormone, which are pivotal hormones for supporting the hypothesis of a primary impairment in testicular function over time.^25^

Recently, Nichols et al published that passive-scatter proton therapy was not associated with testosterone suppression at 5 years, and they suggested that protons may cause less out-of-field scatter radiation than x-rays.^26^ Further prospective studies to directly compare IMRT with other modalities such as brachytherapy, proton therapy, or surgery are warranted.

## Conclusions

In conclusion, TT levels and absolute/relative changes in TT consistently decreased between 3 and 12 months after IMRT. However, the changes were modest and recovered to nearly pretreatment levels at the 24- to 36-month period, except in patients with higher pre-TT levels (4.23 ≤ ng/mL). The difference in TT changes may be partly owed to a variable sensitivity of individual testicular function to scattered radiation during IMRT. Importantly, the proportion of patients with hypogonadal status determined according to TT level did not increase over time until 10 years after IMRT.
